# Greenhouse gas fluxes of different land uses in mangrove ecosystem of East Kalimantan, Indonesia

**DOI:** 10.1186/s13021-024-00263-3

**Published:** 2024-06-01

**Authors:** Virni Budi Arifanti, Randi Ade Candra, Chandra Agung Septiadi Putra, Adibtya Asyhari, Adi Gangga, Rasis Putra Ritonga, Muhammad Ilman, Aji W. Anggoro, Nisa Novita

**Affiliations:** 1 Research Center for Ecology and Ethnobiology, National Research & Innovation Agency of Indonesia (BRIN), Jl. Raya Jakarta Bogor km 46, Cibinong, Bogor, 16911 Indonesia; 2 Yayasan Konservasi Alam Nusantara, Graha Iskandarsyah Bld, 3rd floor, Jl. Iskandarsyah Raya No.66C, Jakarta, 12160 Indonesia

**Keywords:** Mangroves, Aquaculture, Blue carbon, Greenhouse gas, CO_2_, CH_4_, Emissions, Flux, Carbon budget

## Abstract

**Background:**

Mangrove ecosystems exhibit significant carbon storage and sequestration. Its capacity to store and sequester significant amounts of carbon makes this ecosystem very important for climate change mitigation. Indonesia, owing to the largest mangrove cover in the world, has approximately 3.14 PgC stored in the mangroves, or about 33% of all carbon stored in coastal ecosystems globally. Unfortunately, our comprehensive understanding of carbon flux is hampered by the incomplete repertoire of field measurement data, especially from mangrove ecosystem-rich regions such as Indonesia and Asia Pacific. This study fills the gap in greenhouse gases (GHGs) flux studies in mangrove ecosystems in Indonesia by quantifying the soil CO_2_ and CH_4_ fluxes for different land use types in mangrove ecosystems, i.e., secondary mangrove (SM), restored mangrove (RM), pond embankment (PE) and active aquaculture pond (AP). Environmental parameters such as soil pore salinity, soil pore water pH, soil temperature, air temperature, air humidity and rainfall are also measured.

**Results:**

GHG fluxes characteristics varied between land use types and ecological conditions. Secondary mangrove and exposed pond embankment are potential GHG flux sources (68.9 ± 7.0 and 58.5 ± 6.2 MgCO_2_e ha^− 1^ yr^− 1^, respectively). Aquaculture pond exhibits the lowest GHG fluxes among other land use types due to constant inundation that serve as a barrier for the release of GHG fluxes to the atmosphere. We found weak relationships between soil CO_2_ and CH_4_ fluxes and environmental parameters.

**Conclusions:**

The data and information on GHG fluxes from different land use types in the mangrove ecosystem will be of importance to accurately assess the potential of the mangrove ecosystem to sequester and emit GHGs. This will support the GHG emission reduction target and strategy that had been set up by the Indonesian Government in its Nationally Determined Contributions (NDC) and Indonesia’s 2030 Forest and Other Land Use (FOLU) Net Sink.

## Background

Mangroves are the most productive and efficient ecosystem in storing and sequestering carbon in their biomass and sediments [1, 2]. Together with seagrass meadows and saltmarshes, they are known as blue carbon ecosystems, which have a great capacity for carbon sequestration compared to other ecosystems [3]. Mangroves in the Indo-Pacific region were indicated to store three to five times more carbon per unit area compared to tropical terrestrial and boreal forests [1], which makes this ecosystem very important in climate change mitigation and adaptation.

Despite their importance, mangroves worldwide are experiencing significant declines due to large-scale deforestation and conversion to other land uses [4–6]. Once intact mangroves are disturbed and converted to other land uses, they generate substantial greenhouse gas (GHG) emissions [7–12]. Indonesia lost approximately 30% of its mangrove forests between 1980 and 2005 [13], or an estimated GHG emissions of 0.19 Pg CO_2e_ yr^− 1^ [12]. A recent study showed that the mangrove deforestation rate in Indonesia from 2009 to 2019 was estimated at 18,209 ha yr^− 1,^ resulting in total emissions of 1,434,874 Mg CO_2e_ ha^− 1^ yr^− 1^, which comprised approximately 10% of the total projected emissions from the total forestry sector in Indonesia (2006–2020) [6]. Among the regions with the highest mangrove loss in Indonesia, Kalimantan has lost its mangrove cover area of 6,850 ha yr^− 1^ (6,733,941 CO_2e_ yr^− 1^), with the East Kalimantan region being one of the largest areas of mangrove loss in Indonesia, with deforestation rate of 2,007 ha yr^− 1^ (2,529,052 CO_2e_ yr^− 1^) [6]. Although mangroves cover only 2.6% of the total forest area in Indonesia, the mitigation density was four times higher (12.2 MgCO_2e_ ha^− 1^ yr^− 1^) than that of drylands (2.9 MgCO_2e_ ha^− 1^ yr^− 1^ [14]. This highlights the large source of GHG emissions primarily from the soil carbon pool of this ecosystem and underscores the potential emission reduction value of mangroves that can be incorporated into Indonesia’s Nationally Determined Contributions (NDCs) and other climate commitments, such as the Indonesia’s 2030 FOLU Net Sink.

Global estimates of mangrove GHG flux contributions to global GHG emissions are important to assess the potential impacts of mangrove conversion and the potential for GHG mitigation schemes [15, 16]. Although studies on carbon stocks and GHG fluxes have been conducted over the past decades, there is still limited carbon flux research across the Asia-Pacific region, including Indonesia [15]. Specifically, there are very limited empirical studies on GHG emissions from mangrove converted aquaculture ponds, which is the most important driver of mangrove loss in the tropics.

Specifically, various land cover conditions neighboring mangrove ecosystems may have different carbon stocks and fluxes [8, 12, 16–20], resulting in different GHG mitigation schemes. For instance, soil CO_2_ fluxes in aquaculture ponds (23.8 MgCO_2e_ ha^− 1^ yr^− 1^) are three times higher than those in undisturbed mangroves (7.9 MgCO_2e_ ha^− 1^ yr^− 1^) [20]. Although well studied, detailed temporal monitoring of GHG fluxes is needed to better understand the dynamics of carbon sources and sinks across different land covers, which will impact land management.

To elucidate the fate of mangrove blue carbon as a source and sink, more data on mangrove blue carbon stocks and fluxes [15] are needed for different management regimes. Therefore, the aims of this study are to examine the GHG fluxes in several mangrove land use types, such as secondary mangrove, restored mangrove, aquaculture pond and pond embankment, and analyze the relationships between the environmental parameters and GHG fluxes. Ultimately, this study is important to understand the environmental factors that might influence the GHG fluxes in mangrove land cover types and to fill the gaps in the limited GHG flux data in mangrove ecosystems. This information is important to provide accurate information for the national climate mitigation targets that had been outlined in the Indonesia’s Nationally Determined Contributions (NDC) and Forest and Other Land Use (FOLU) Net Sink 2030.

## Method

### Study site

This study was conducted in a mangrove ecosystem in Tabalar Muara Village, Berau Regency, East Kalimantan, Indonesia. This area had been converted to aquaculture in the past and is currently being restored under an initiative called the “Shrimp-Carbon Aquaculture (SECURE)” introduced by Yayasan Konservasi Alam Nusantara (YKAN). This initiative aims to restore the mangrove ecosystem and increase the traditional shrimp production by narrowing the aquaculture area to 20% of its original size and utilizing the remaining 80% for mangroves.

**Fig. 1:**
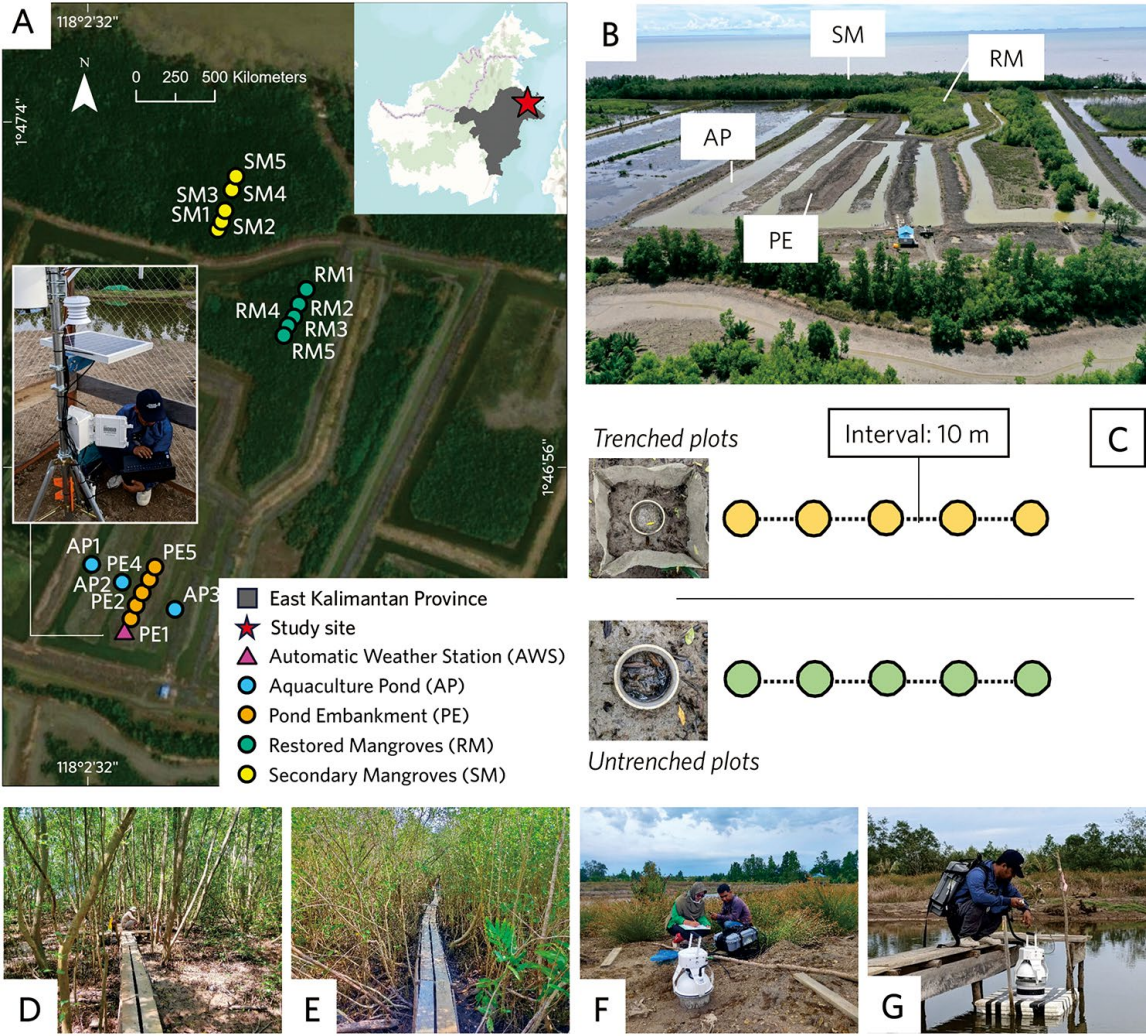
Study site with the plot layout in Tabalar Muara Village, Berau Regency, East Kalimantan; (**A**) monitoring sites for all four land use types; (**B**) aerial view of the study area; (**C**) plot layout of untrenched and trenched plots for measuring the total (Rt) and heterotrophic (Rh) respiration, respectively; (**D**) secondary mangrove; (**E**) restored mangrove; (**F**) pond embankment; (**G**) aquaculture pond

The measurements were conducted in a secondary mangrove (SM), restored mangrove (RM), pond embankment (PE) and active aquaculture pond (AP). Pond embankment and aquaculture pond exist along the year, they do not experience tides since the water is controlled by a water gate. Secondary mangrove is a mangrove forest that has been disturbed due to the development of aquaculture ponds in the past, has been undergoing natural regeneration to form secondary mangroves and is influenced by tides (Fig. 1D). Restored mangrove consists of natural regrowth on aquaculture ponds and is influenced by the water management of the ponds’ sluices or water gate (Fig. 1E). The pond embankment consists of dikes built surrounding aquaculture ponds (Fig. 1F). Embankments are considered to be different from active aquaculture ponds due to the characteristics they possess, e.g., bare land, dry (never inundated by water) and compact soils. An aquaculture pond is an active pond where aquaculture production is still in place (Fig. 1G).

### Field sampling

In secondary and restored mangroves, we made each a linear transect consisting of ten measurement plots, by which five plots were trenched (in 10 m intervals) and five other plots were untrenched (Fig. 1C). Trenched and untrenched plots were used to measure heterotrophic (Rh) and total respiration (Rt), respectively. The 1 × 1 m^2^ trenches were made of aluminum mesh sheets and were inserted at the minimum of 60 cm into the soil. The dominant vegetation in SM and RM is *Avicennia* spp., with shallow surface pencil roots. Here we assume that the mangrove roots are less than 60 cm deep.

The distribution and placement of the plots were designated considering the spatial variability and practicality of the measurements. In the aquaculture pond, we made five plots on the pond’s embankment and three plots on the water of the active pond using a modified chamber placed on a floating buoy (Fig. 1G). In PE there is no vegetation so we only measured soil respiration that is predominantly generated from microbial decomposition (Rh), with the same number of Rh plots as in SM and RM. In AP the sampling points are chosen to capture the variability of the pond’s depth and considering the practical reasons where we have to build a wooden bridge above the water. Wooden boardwalks/bridges were built along the transects of each land cover type to avoid soil compaction before and during measurements (Fig. 1D, E).

GHG fluxes were measured starting one month after the permanent chamber bases and boardwalks had been established in the study sites. An automatic weather station (HOBO U30-NRC) was installed within the research site to measure air temperature, air humidity, air pressure and precipitation (Fig. 1A). Cumulative precipitation was calculated on a monthly basis. The weather station is considered representative to cover the whole research site, where the distance of the furthest plot (SM) is approximately 500 m from the location of the automatic weather station.

The CO_2_ and CH_4_ fluxes and environmental parameters such as soil pore water salinity, soil pore water pH, soil temperature, air temperature and humidity were measured at different land use types during three consecutive days on a monthly basis for 6 months from October 2022 to March 2023. The soil temperature was measured at approximately 10 cm deep in each plot. Our study was conducted during the rainy season considering the high potential of soil GHG fluxes arising from tropical mangrove forests in the wet season [21–24]. In addition, during 2000–2023 we have been experiencing La Nina resulting in high precipitation rates and wetter conditions along the year.

Surface water temperature was measured at 5 cm depth in the aquaculture pond since the water quality instrument could not reach the soil beneath the pond. Three consecutive days of GHG fluxes measurements were treated as replications to increase the accuracy of the measured monthly dataset. In this study, the GHGs and other environmental parameters were measured during low tides at daytime in three consecutive days. Many studies on GHGs measurements had been conducted on daytime during low tides [24–27], which are mainly due to safety reasons and to avoid risks inside the forests at nighttime [27]. We made sure that the tidal range, tidal period, exposure duration, measurement time and the meteorological condition, were comparable among sampling days in all sites. In general, we assume the microbial activities are relatively the same during the time of measurements.

The CO_2_ and CH_4_ fluxes were measured using a LI-COR LI-7810 trace gas analyzer equipped with 8200–01 S Smart Chamber from LI-COR (Fig. 1F, G). The 8200–01 S Smart Chamber is a brand for chamber products from LI-COR, which consists of a portable, battery-powered chamber with an embedded microprocessor and internal storage for real-time flux calculations with a LI-COR gas analyzer. An automated mechanism seals the chamber around the permanent collar that had been placed one month before the initial measurement which ensures the isolation of the gases inside the chamber. Any soil disturbance and fluxes are also minimally affected because the chamber never touches the collar directly [28]. The CH_4_ flux was converted to CO_2_ equivalent by applying its global warming potential (GWP) over a 100-year period of 27.2 according to the IPCC Sixth Assessment Report [29]. Air temperature and humidity were acquired from the static automatic weather station installed in the field. Soil temperature and soil moisture was measured using a Stevens HydraProbe that comes equipped with the LI-COR chamber. Soil pore salinity and pH were acquired from EZ-9909 portable water quality monitoring instrument.

### Data analysis

Differences in GHG fluxes and other environmental parameters among land use types were assessed utilizing analysis of variance (ANOVA) [30]. Tukey’s and Games-Howell honest significant difference post hoc tests were applied to determine the significance of means when the ANOVA result was significant [30]. Data normality was analyzed based on the Kolmogorov‒Smirnov and Shapiro‒Wilk tests. A t-test was applied to determine the difference between two normally distributed dataset. A significance test was applied between the trenched (Rh) and untrenched (Rt) plots for each land use type using a t-test. One-way ANOVA was performed to analyze the differences between the mean values of more than two land use types.

The significance test of the environmental parameters was analyzed using one-way ANOVA. For non-homogeneous variances (i.e., soil pore water salinity, pH and soil temperature), the Games-Howell post hoc test was applied. For homogeneous parameters (i.e., air temperature and humidity), the Bonferroni post hoc correction was applied. We used Pearson’s correlation analysis to assess the linear relationship between environmental variables using the following formula [30, 31]:

$$\rho \,X,Y\, = \,{{{\mathop{\rm cov}} \,(X,\,Y)} \over {\sigma \,X\,\sigma \,Y}}$$

Where ρ is the Pearson correlation coefficient of population *X* and *Y*, cov is the covariance, σ *X* is the standard deviation of *X*, and σ *Y* is the standard deviation of *Y*.

Statistical analyses were conducted using Microsoft Excel and IBM SPSS Statistics 22.0.

## Results

### CO_2_, CH_4_, and total GHG fluxes

The mean CO_2_, CH_4_ and total GHG fluxes were calculated for each land use type (Table 1). The total GHG flux is the sum of CO_2_ and CH_4_ fluxes.

**Table 1:** CO_2_, CH_4_ and total GHG fluxes of different land use types. Values are the mean ± SE

Land use	CO_2_ flux (MgCO_2_e ha^− 1^ yr^− 1^)	CH_4_ flux (MgCO_2_e ha^− 1^ yr^− 1^)	Total GHG flux (MgCO_2_e ha^− 1^ yr^− 1^)
Secondary mangrove (SM)	36.9 ± 3.4	32.0 ± 5.7	68.9 ± 7.0
Restored mangrove (RM)	28.0 ± 2.1	0.3 ± 0.1	28.3 ± 2.1
Pond’s embankment (PE)	52.8 ± 4.8	5.6 ± 2.7	58.5 ± 6.2
Aquaculture pond (AP)	2.9 ± 0.4	0.3 ± 0.02	3.2 ± 0.4

CO_2_ fluxes varied between land use types at the Muara Tabalar site. The pond embankment had the highest CO_2_ flux (52.8 ± 4.8 MgCO_2_e ha^− 1^ yr^− 1^), which significantly differed from other land use types (*p* < 0.01), followed by secondary mangroves (36.9 ± 3.4 MgCO_2_e ha^− 1^ yr^− 1^) and restored mangroves (28.0 ± 2.1 MgCO_2_e ha^− 1^ yr^− 1^). Active pond had the lowest CO_2_ flux (2.9 ± 0.4 MgCO_2_e ha^− 1^ yr^− 1^) (Table 1).

The highest CH_4_ flux was observed in the secondary mangrove (32.0 ± 5.7 MgCO_2_e ha^− 1^ yr^− 1^), which was significantly different from other land use types (*p* < 0.01), followed by pond embankment (5.6 ± 2.7 MgCO_2_e ha^− 1^ yr^− 1^). The lowest CH_4_ flux was found in active aquaculture pond (0.3 ± 0.02 MgCO_2_e ha^− 1^ yr^− 1^) and restored mangrove (0.3 ± 0.1 MgCO_2_e ha^− 1^ yr^− 1^).

### Total and heterotrophic respiration

The total (Rt) and heterotrophic (Rh) soil respiration were measured from untrenched and trenched plots, respectively, for each land use type (Table 2).

**Table 2:** The total and heterotrophic respiration of secondary and restored mangroves (Rh: heterotrophic respiration; Rt: total respiration). Values are the mean ± SE

Land use	Respiration	CO_2_ flux (MgCO_2_e ha^− 1^ yr^− 1^)
Secondary mangroves (SM)	Rh	22.1 ± 2.7
	Rt	36.9 ± 3.4
Restored mangroves (RM)	Rh	29.6 ± 2.3
	Rt	28.0 ± 2.1

The highest mean of the total soil CO_2_ flux (Rt) was found in the secondary mangroves (36.9 ± 3.4 MgCO_2_e ha^− 1^ yr^− 1^) (Table 2). There was a significant difference in the CO_2_ flux between Rh and Rt in the secondary mangrove (*p* = 0.001). There were no significant differences between the CO_2_ flux of the heterotrophic and total respiration in the restored mangrove (*p* = 0.6). We found significant differences between the Rt and Rh of the CO_2_ fluxes in the secondary and restored mangroves (*p* = 0.03 and *p* = 0.04, respectively).

### Environmental parameters

Environmental parameters such as soil pore water salinity, soil pore water pH, soil temperature, air temperature and humidity show varied trends in different land use types. The highest soil pore salinity was found in active aquaculture pond, followed by secondary mangrove, restored mangrove and pond embankment (Table 3).

**Table 3:** Environmental parameters (soil pore water salinity, pH, soil temperature, air temperature and humidity) of different land use types. In AP the surface water salinity and temperature were measured instead of soil pore water salinity and soil temperature. The values represent the mean ± SE

Environmental parameters	Soil pore water salinity (ppt)	pH	Soil temperature (°C)	Air temperature (C)	Air humidity (%)
Secondary mangroves (SM)	14.7 ± 1.0	6.7 ± 0.2	30.2 ± 0.1	30.4 ± 0.6	63.9 ± 1.3
Restored mangroves (RM)	6.8 ± 0.7	6.5 ± 0.2	31.18 ± 0.2	31.4 ± 0.6	60.1 ± 1.3
Pond embankment (PE)	1.5 ± 0.1	3.5 ± 0.1	32.0 ± 0.3	31.3 ± 1.4	62.0 ± 1.9
Aquaculture pond (AP)	17.0 ± 1.5*	6.9 ± 0.3	31.1 ± 0.3*	30.4 ± 1.0	64.9 ± 2.3

*surface water

There were no significant differences in the mean soil pore water pH values between secondary, restored mangrove and aquaculture ponds. However, the pH values of these land uses differ significantly from those of the pond embankment (*p* < 0.0001).

The mean soil temperature of the different land use types ranges from 30.2 to 32.0 **°**C. We found significant differences in the mean soil temperatures between secondary mangrove, restored mangrove and pond embankment.

Air temperature ranges from 30.4 to 31.4 **°**C for all land use types, with the highest value being observed in restored mangrove (31.4 **°**C). There are no significant differences between the air temperature in all land use types. Air humidity ranges from 60.1 to 64.9% for all land use types, with no significant differences between land use types (*p =* 0.1).

Precipitation was measured for the whole study site during the rainy season (October 2022 - March 2023), with a mean monthly rainfall of 228.8 mm month^− 1^. The highest rainfall was measured in March 2023 (389.9 mm), while the lowest was observed in December 2022 (139 mm).

The correlations between the soil CO_2_ and CH_4_ fluxes and environmental parameters such as soil pore salinity, pH, soil temperature, air temperature and humidity were analyzed using Pearson correlation (Table 4).

**Table 4:** Pearson correlation of soil CO_2_ and CH_4_ fluxes with the environmental parameters

		Soil pore salinity	pH	Soil temperature	Air temperature	Air humidity	Rainfall
CO_2_	Pearson Correlation	-0.289**	-0.150**	-0.03	0.132**	0.139**	0.049
	Sig. (2-tailed)	0	0.001	0.506	0.003	0.002	0.270
CH_4_	Pearson Correlation	0.209**	0.095*	-0.121**	-0.148**	-0.073	-0.087*
	Sig. (2-tailed)	0	0.034	0.007	0.001	0.101	0.050

*. Correlation is significant at the 0.05 level (2-tailed)

**. Correlation is significant at the 0.01 level (2-tailed)

There is a significant correlation between the soil CO_2_ flux and soil pore salinity, pH, air temperature and air humidity (Table 4). Although the Pearson correlations for these parameters were significant, the regression analysis for the soil pore water salinity, pH and soil temperature relationship showed low R^2^ values (0.084; 0.022; 0.018 and 0.019, respectively). This implies a weak relationship between the soil CO_2_ flux and other environmental parameters.

Significant correlations are also found between the soil CH_4_ flux and soil pore water salinity, pH, soil, air temperature and rainfall (Table 4). Soil pore water salinity and pH showed positive correlations with soil CH_4_ flux. In contrast, soil and air temperature, air humidity and rainfall exhibit negative correlations. However, the low R^2^ values exhibited by these parameters indicate a very weak relationship between the CH_4_ flux and these parameters (R^2^ = 0.005, 0.015, 0.022 and 0.002, respectively).

**Fig. 2:**
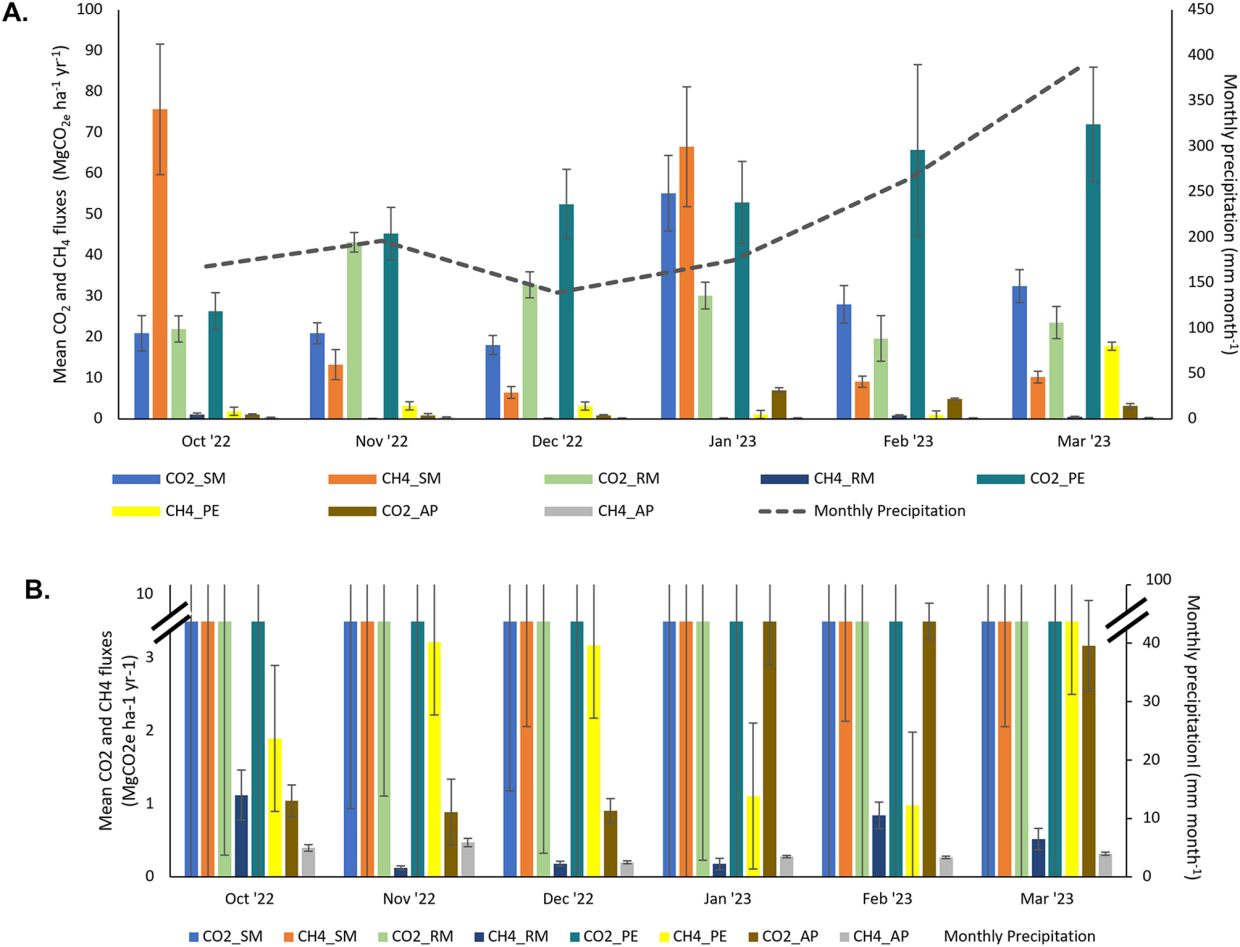
(**A**) CO_2_ and CH_4_ fluxes (values are mean ± SE) from all land use types and monthly precipitation (mm month^− 1^) during the study period; (**B**) Magnification of the CH_4_ fluxes from all land use types during the study period

Generally, monthly CO_2_ fluxes for all land use types were lower during the transition of the dry season to the start of the rainy season (October - December) and showed a gradual increase toward the peak of the rainy season (January - March) (Fig. 2A). The CO_2_ flux in the pond embankment showed a steady increase during the observation period and reached its peak in March, while the secondary mangrove, restored mangrove and aquaculture pond had fluctuating CO_2_ fluxes throughout the study period.

Unlike the CO_2_ flux, the monthly mean of the CH_4_ flux does not exhibit a certain seasonal trend (Fig. 2B). Secondary mangrove predominantly had the highest CH_4_ flux compared to other land use types across seasons, followed by the pond embankment, restored mangrove and aquaculture pond.

## Discussion

### CO_2_, CH_4_, and total GHG fluxes

This study shows that dry pond embankment serves as a significant source of CO_2_ fluxes compared to other land uses, followed by secondary mangrove, restored mangrove and aquaculture pond (Table 1). Our result is in line with the findings from the average soil CO_2_ flux in aquaculture ponds which is about three times higher than the undisturbed mangroves in Indonesia [20]. Generally, the CO_2_ flux observed in the pond embankment in this study (52.8 ± 4.8 MgCO_2_e ha^− 1^ yr^− 1^) is higher than the range of the CO_2_ flux measurements on the abandoned ponds in Bali and Sulawesi (15.9–43.7 MgCO_2_e ha^− 1^ yr^− 1^) [32, 33]. The CH_4,_ flux in the pond embankment from this study (5.6 MgCO_2_e ha^− 1^ yr^− 1^) is twice higher than the exposed pond in South Sulawesi, Indonesia (2.5 MgCO_2_e ha^− 1^ yr^− 1^) [32].

The total GHG fluxes, including CO_2_ and CH_4,_ fluxes in the secondary mangrove and pond embankment have the highest values compared to other land uses. In the secondary mangrove, there is an almost equal amount of CO_2_ and CH_4_ fluxes, while in the pond’s embankment, the dominant source of GHG was derived from CO_2_ flux (90% of the total GHG flux in the pond’s embankment). The relatively high CO_2_ flux from the pond’s embankment might be impacted by the amount of organic matter and nutrients from wastewater, shrimp pond effluent and residual feeds deposited in the dikes [34, 35]. However, more detailed study should be conducted on the impacts of the organic matter and nutrients to CO_2_ flux as we did not measure these parameters in this study. Moreover, the pond’s embankment, which is exposed to direct sunlight, results in high rates of organic material decomposition deposited in the dikes and becoming the dominant source of soil respiration [2]. Higher temperatures in the soils of pond embankments (Table 3) could also increase organic matter decomposition from microbial activity, consequently resulting in higher CO_2_ flux released to the atmosphere [36–38].

Our results show that secondary mangroves are potential CO_2_ and CH_4_ sources. The presence of mangrove trees in the secondary mangrove with their pneumatophores serve as conduits of GHG fluxes to the atmosphere. The mean CO_2_ fluxes of the secondary and restored mangroves in this study are twice as high as those of the secondary mangroves in eastern Thailand (6.9–12.5 MgCO_2_e ha^− 1^ yr^− 1^) [39]. There is a distinct difference in soil temperature between our study site and the study site in eastern Thailand [39]. The mean soil temperatures of the secondary and restored mangroves in this study (ranging from 30.2 to 31.18 °C) are higher than in Thailand sites (ranging from 27.71 to 30.14 °C). Secondary and restored mangroves have significantly higher CO_2_ fluxes which resulted from mangrove root respiration and the high rates of primary production deriving from litterfall forming carbon detritus for microbes [40, 41].

The secondary mangrove, which is located adjacent to the Sulawesi Sea, is experiencing a regular tidal cycle and is periodically inundated by tides. CH_4_ is produced under anaerobic condition during high tides when the mangrove soil is inundated, but CH_4_ cannot be released to the atmosphere due to the water barrier. The ebbing of the tides will remove the water barrier and release the CH_4_ produced by the soil to the atmosphere [42, 43]. This tidal influence on the CH_4_ flux is therefore reported to generate the highest flux at mid to low tide when the gas concentrations in the water are highest and the sediments are exposed [44]. Although we did not specifically measure the density of the roots and crab burrows, studies have reported that the total CH_4_ emissions from the mangrove sediments may also be enhanced by the flux generated by the pneumatophores, roots and crab burrows [21].

Although the CH_4_ flux of the secondary mangrove is significantly high compared to other studies, the CH_4_ flux of the restored mangrove in this study (0.3 MgCO_2_e ha^− 1^ yr^− 1^) is similar compared to other studies, e.g., Ayeyarwady Delta, Myanmar (0.2–0.3 MgCO_2_e ha^− 1^ yr^− 1^) [17] and North Sulawesi, Indonesia (0.4 MgCO_2_e ha^− 1^ yr^− 1^) [32] and ranged in the lower end compared to the Cauvery Delta, India (1.9–3.7 MgCO_2_e ha^− 1^ yr^− 1^) [45].

Active aquaculture pond that was constantly inundated had the lowest CO_2_, CH_4_ and total soil GHG fluxes (3.2 ± 0.4 MgCO_2_e ha^− 1^ yr^− 1^) compared to other land uses (Table 1). The CO_2_ flux from active pond in this study (2.9 MgCO_2_e ha^− 1^ yr^− 1^) was higher than in South Sulawesi (0.5 MgCO_2_e ha^− 1^ yr^− 1^), while the CH_4_ flux in this study (0.3 MgCO_2_e ha^− 1^ yr^− 1^) was somewhat lower than that in South Sulawesi (0.6 MgCO_2_e ha^− 1^ yr^− 1^) [46]. The low GHG fluxes from aquaculture pond might be due to constant water inundation that serves as a barrier for GHG fluxes to be released to the atmosphere. However, it can be counterbalanced by the GHG emissions arising from land use change due to dredging during conversion of mangroves to aquaculture pond, resulting in aquaculture ponds becoming net GHG sources as what had been occurred in the Mahakam Delta, East Kalimantan, Indonesia [8, 16, 47].

### Total and heterotrophic respiration

Total soil respiration (Rt) is the total production of CO_2_ at the soil surface, which is the sum of autotrophic and heterotrophic respiration [48]. Heterotrophic respiration is predominantly generated from the decomposition of organic materials by bacteria [49]. In secondary mangrove, we found that approximately 60% of the soil respiration comes from organic matter decomposition by microbial activities (Rh). In contrast, higher heterotrophic respiration than the total respiration was found in restored mangrove (Table 2). Several studies [35, 39] have suggested attributing the total soil respiration (Rt) to heterotrophic respiration (Rh) based upon the assumption that belowground root respiration is primarily released through aboveground roots (e.g., prop roots, pneumatophores). Therefore, more in-depth research should be conducted to determine the role and contributions of mangrove’s belowground roots to the total soil flux (Rt).

### Environmental parameters

There were highly significant differences between the soil pore water salinity of the secondary mangrove and active aquaculture pond and between the restored mangrove and pond embankment (*p* < 0.01). Compared to soil pore water salinity data from other sites in Indonesia (i.e., the Mahakam Delta, East Kalimantan) [47], the salinity from the different land use types in this study is quite low. This might be due to the lack of tidal influences on these land use types in Tabalar Muara village, as the water entering the restored mangrove and pond is controlled by the sluice gates. Low mean salinity in the secondary mangrove of this study (14.7 ppt) may result in relatively high CH_4_ flux (32.7 MgCO_2_e ha^− 1^ yr^− 1^) compared to other studies [17, 32, 45].

The mean pH values in secondary and restored mangroves, and aquaculture pond are quite high (6.5–6.9), which might indicate that the soil water in these land uses is brackish. In contrast, the low pH in the pond’s embankment (3.5) may indicate the acidification process that previously had taken place in this plot.

The significant differences in the soil temperature between the land use types indicate that the effect of vegetation cover and water inundation is important to provide a cooling effect for the soils. This was shown in this study, where the secondary mangrove, which experiences a regular tidal cycle and has a closed canopy cover, exhibits the lowest soil temperature, while the pond embankment with exposed dry soil has the highest soil temperature (Table 3). However, there are no significant differences in the air temperature and humidity between the different land use types.

The inverse correlation between soil CO_2_ flux and soil pore water salinity, pH, and soil temperature in this study may imply that the increase in soil pore salinity, pH, and soil temperature will decrease the soil CO_2_ flux. In contrast, lower soil pore water salinity, pH, and soil temperature result in higher soil CO_2_ flux. However, these relationships are weak due to the low R^2^ values exhibited by these parameters. Low salinity and pH might favor microbial activities, resulting in increased GHG emissions, but the effect of low soil temperature on increased GHG emissions is not commonly found.

The significant and positive correlation between the soil CH_4_ flux and soil pore water salinity and pH indicate that there is a tendency for CH_4_ flux to increase with soil pore water salinity and pH (Table 4). However, methanogenesis is usually inhibited by high sulfate concentrations in seawater, and some studies have reported that methanogenesis could be found in estuarine forests with low salinities [50]. The weak relationship between soil CH_4_ flux and soil pore water salinity is indicated by low R^2^ (0.044).

The CH_4_ flux decrease is in line with higher air humidity, precipitation, soil and air temperature. This inverse correlation could be explained by the methanogenesis being heightened when there is a lack of O_2_ [50]; in contrast, higher temperature and humidity resulting from soil exposure due to solar radiation could suppress CH_4_ emissions. The fact that the observation period was conducted only during the rainy season might need to be expanded to the whole year observation to obtain a complete seasonal variance of the gas fluxes and other environmental parameters.

## Conclusion

Secondary mangroves and pond embankment soils may serve as potential GHG (CO_2_ and CH_4_ fluxes) sources. We found that pond embankments are the dominant source of CO_2_ flux due to the dry and exposed condition leading to high rates of organic matter decomposition by microbial activities. Secondary mangrove may become a potential CH_4_ flux source due to methanogenesis occurring during high tides which will be released to the atmosphere when the tide recedes. Active aquaculture ponds exhibit the lowest GHG flux due to water impoundment, which can hamper the release of CO_2_ to the atmosphere and the limited organic materials being decomposed by microbial activities. Although aquaculture ponds may serve as weak carbon sinks, the CO_2_ emissions generated from land use changes during mangrove conversion to aquaculture ponds may result in significant CO_2_ emissions [8, 16] that need to be considered in future GHG assessments. Therefore, a comprehensive carbon cycle analysis, especially during the first years of mangrove conversion, is strongly advised to obtain accurate information on the impacts of different mangrove management practices on climate mitigation.

## References

[1] Donato DC, Kauffman JB, Murdiyarso D, Kurnianto S, Stidham M, Kanninen M. Mangroves among the most carbon-rich forests in the tropics. Nat Geosci [Internet]. 2011 Apr 3 [cited 2014 Jan 21];4(5):293–7. http://www.nature.com/doifinder/10.1038/ngeo1123.

[2] Kristensen E, Bouillon S, Dittmar T, Marchand C. Organic carbon dynamics in mangrove ecosystems: A review. Aquat Bot [Internet]. 2008 Aug [cited 2014 Jan 21];89(2):201–19. http://linkinghub.elsevier.com/retrieve/pii/S0304377007001817.

[3] Mcleod E, Chmura GL, Bouillon S, Salm R, Björk M, Duarte CM et al. A blueprint for blue carbon: toward an improved understanding of the role of vegetated coastal habitats in sequestering CO 2. Front Ecol Environ [Internet]. 2011 Dec [cited 2014 Apr 28];9(10):552–60. 10.1890/11000410.1890/110004.

[4] Hamilton SE, Casey D (2016). Creation of a high spatio-temporal resolution global database of continuous mangrove forest cover for the 21st century (CGMFC-21). Glob Ecol Biogeogr.

[5] Richards DR, Friess DA. Rates and drivers of mangrove deforestation in Southeast Asia, 2000–2012. Proc Natl Acad Sci [Internet]. 2016;113(2):344–9. http://www.pnas.org/lookup/doi/10.1073/pnas.1510272113. 10.1073/pnas.1510272113 PMC4720307 26712025

[6] Arifanti VB, Kauffman JB, Subarno, Ilman M, Tosiani A, Novita N (2022). Contributions of mangrove conservation and restoration to climate change mitigation in Indonesia. Glob Chang Biol.

[7] Hamilton SE, Friess DA (2018). Global carbon stocks and potential emissions due to mangrove deforestation from 2000 to 2012. Nat Clim Chang.

[8] Kauffman JB, Arifanti V, Trejo H, Jesús García M, del Norfolk C, Hadriyanto J. D, The jumbo carbon footprint of a shrimp: carbon losses from mangrove deforestation. Front Ecol Environ. 2017.

[9] Atwood TB, Connolly RM, Almahasheer H, Carnell PE, Duarte CM, Lewis CJE (2017). Global patterns in mangrove soil carbon stocks and losses. Nat Clim Chang.

[10] Arifanti VB, Novita N, Subarno, Tosiani A. Mangrove deforestation and CO_2_ emissions in Indonesia. IOP Conf Ser Earth Environ Sci. 2021;874(1).

[11] Arifanti VB, Kauffman JB, Hadriyanto D, Murdiyarso D, Diana R. Carbon dynamics and land use carbon footprints in mangrove-converted aquaculture: The case of the Mahakam Delta, Indonesia. For Ecol Manage [Internet]. 2019;432(January 2019):17–29. https://linkinghub.elsevier.com/retrieve/pii/S0378112718301427.

[12] Murdiyarso D, Purbopuspito J, Kauffman JB, Warren MW. The potential of Indonesian mangrove forests for global climate change mitigation-supplementary. 2015;2010(2):1–10.

[13] FAO. The world’s mangroves 1980–2005. Vol. 153, FAO Forestry Paper. 2007.

[14] Novita N, Subarno, Lestari NS, Anshari GZ, Lugina M, Yeo S et al. Natural climate solutions in Indonesi: wetlands are the key to achieve Indonesia’s national climate commitment. Environ Res Lett. 2022;17.

[15] Sharma S, Ray R, Martius C, Murdiyarso D (2023). Carbon stocks and fluxes in Asia-Pacific mangroves: current knowledge and gaps. Environ Res Lett.

[16] Arifanti VB, Kauffman JB, Hadriyanto D, Murdiyarso D, Diana R. Carbon dynamics and land use carbon footprints in mangrove-converted aquaculture: the case of the Mahakam Delta, Indonesia. Ecol Manage. 2019;432.

[17] Cameron C, Hutley LB, Friess DA, Brown B (2019). Community structure dynamics and carbon stock change of rehabilitated mangrove forests in Sulawesi, Indonesia Community structure dynamics and carbon stock change of rehabilitated mangrove forests in Sulawesi, Indonesia. Ecol Appl.

[18] Sasmito SD, Taillardat P, Clendenning J, Friess DA, Murdiyarso D, Hutley LB. Carbon stocks and fluxes associated with land-use and land-cover change in mangrove ecosystems: A systematic review protocol. Bogor, Indonesia; 2016. Report No.: 211.

[19] Sasmito SD, Taillardat P, Clendenning JN, Cameron C, Friess DA, Murdiyarso D (2019). Effect of land-use and land-cover change on mangrove blue carbon: a systematic review. Glob Chang Biol.

[20] Murdiyarso D, Krisnawati H, Adinugroho WC, Sasmito SD. Deriving emission factors for mangrove blue carbon ecosystem in Indonesia. Carbon Balance Manag [Internet]. 2023;18(12). 10.1186/s13021-023-00233-1. 10.1186/s13021-023-00233-1 PMC10339514 37439912

[21] Kristensen E, Flindt MR, Ulomi S, Borges AV, Abril G, Bouillon S (2008). Emission of CO2 and CH4 to the atmosphere by sediments and open waters in two Tanzanian mangrove forests. Mar Ecol Prog Ser.

[22] Otero XL, Araújo JMC, Barcellos D, Queiroz HM, Romero DJ, Nóbrega GN (2020). Crab bioturbation and seasonality control nitrous oxide emissions in semiarid mangrove forests (Ceará, Brazil). Appl Sci.

[23] Kitpakornsanti K, Pengthamkeerati P, Limsakul A, Worachananant P, Diloksumpun S. Greenhouse gas emissions from soil and water surface in different mangrove establishments and management in Ranong Biosphere Reserve, Thailand. Reg Stud Mar Sci [Internet]. 2022;56:102690. 10.1016/j.rsma.2022.102690.

[24] Sugiana IP, Faiqoh E, Adame MF, Indrawan GS, Andiani AAE, Dewi IGAIP et al. Soil greenhouse gas fluxes to the atmosphere during the wet season across mangrove zones in Benoa Bay, Indonesia. Asian J Atmos Environ [Internet]. 2023;17(1). 10.1007/s44273-023-00014-9.

[25] Chen GC, Tam NFY, Ye Y. Summer fluxes of atmospheric greenhouse gases N2O, CH4 and CO2 from mangrove soil in South China. Sci Total Environ [Internet]. 2010;408(13):2761–7. 10.1016/j.scitotenv.2010.03.007. 10.1016/j.scitotenv.2010.03.007 20381125

[26] Chen GC, Ulumuddin YI, Pramudji S, Chen SY, Chen B, Ye Y et al. Rich soil carbon and nitrogen but low atmospheric greenhouse gas fluxes from North Sulawesi mangrove swamps in Indonesia. Sci Total Environ [Internet]. 2014;487:91–6. 10.1016/j.scitotenv.2014.03.140. 10.1016/j.scitotenv.2014.03.140 24784732

[27] Chanda A, Akhand A, Manna S, Dutta S, Das I, Hazra S et al. Measuring daytime CO2 fluxes from the inter-tidal mangrove soils of Indian Sundarbans. Environ Earth Sci [Internet]. 2014 Nov 30 [cited 2015 Jan 22];72(2):417–27. http://link.springer.com/10.1007/s12665-013-2962-2.

[28] LI-COR. Soil gas flux measurement solutions [Internet]. LI-COR. https://www.licor.com/env/products/soil_flux/?gclid=EAIaIQobChMIvITAwae86wIVk6yWCh3NgQW5EAAYASAAEgK4G_D_BwE.

[29] Smith C, Nicholls RJ, Armour K, Collins W, Forster P, Meinshausen M et al. IPCC AR6 WGI Chap. 7: The Earth’s energy budget, climate feedbacks, and climate sensitivity - Supplementary Material. Climate Change 2021: The Physical Science Basis. Contribution of Working Group I to the Sixth Assessment Report of the Intergovernmental Panel on Climate Change. 2021.

[30] Ramsey FL, Schafer DW (2013). The statistical sleuth: a course in methods of Data Analysis.

[31] Blyth S (1994). Karl Pearson and the correlation curve. Int Stat Rev.

[32] Cameron C, Hutley LB, Friess DA, Munksgaard NC. Hydroperiod, soil moisture and bioturbation are critical drivers of greenhouse gas fluxes and vary as a function of landuse change in mangroves of Sulawesi, Indonesia. Sci Total Environ [Internet]. 2019;654(1):365–77. 10.1016/j.scitotenv.2018.11.092. 10.1016/j.scitotenv.2018.11.092 30447576

[33] Sidik F, Lovelock CE. CO_2_ efflux from shrimp ponds in Indonesia. PLoS One [Internet]. 2013 Jan [cited 2014 May 9];8(6):e66329. http://www.pubmedcentral.nih.gov/articlerender.fcgi?artid=3674011&tool=pmcentrez&rendertype=abstract 10.1371/journal.pone.0066329 PMC3674011 23755306

[34] Chen GC, Tam NFY, Wong YS, Ye Y (2011). Effect of wastewater discharge on greenhouse gas fluxes from mangrove soils. Atmos Environ.

[35] Chen G, Chen B, Yu D, Tam NFY, Ye Y, Chen S. Soil greenhouse gas emissions reduce the contribution of mangrove plants to the atmospheric cooling effect. Environ Res Lett. 2016;11(12).

[36] Lovelock CE, Ruess RW, Feller IC. CO2 efflux from cleared mangrove peat. PLoS One [Internet]. 2011 Jan [cited 2014 Dec 15];6(6):e21279. http://www.pubmedcentral.nih.gov/articlerender.fcgi?artid=3126811&tool=pmcentrez&rendertype=abstract 10.1371/journal.pone.0021279 PMC3126811 21738628

[37] Pendleton L, Donato DC, Murray BC, Crooks S, Jenkins WA, Sifleet S et al. Estimating global blue carbon emissions from conversion and degradation of vegetated coastal ecosystems. PloS onePloS one [Internet]. 2012;7(9):e43542. http://journals.plos.org/plosone/article?id=10.1371/journal.pone.0043542. 10.1371/journal.pone.0043542 PMC3433453 22962585

[38] Raich JW (2000). Tufekciogul a. vegetation and soil respiration: correlations and controls [review]. Biogeochemistry.

[39] Poungparn S, Komiyama A, Tanaka A, Sangtiean T, Maknual C, Kato S (2009). Carbon dioxide emission through soil respiration in a secondary mangrove forest of eastern Thailand. J Trop Ecol.

[40] Sidik F, Adame MF, Lovelock CE. Carbon sequestration and fluxes of restored mangroves in abandoned aquaculture ponds. J Indian Ocean Reg [Internet]. 2019;00(0):1–16. 10.1080/19480881.2019.1605659.

[41] Cameron C, Hutley LB, Munksgaard NC, Phan S, Aung T, Thinn T et al. Impact of an extreme monsoon on CO2 and CH4 fluxes from mangrove soils of the Ayeyarwady Delta, Myanmar. Sci Total Environ [Internet]. 2021;760(November):143422. 10.1016/j.scitotenv.2020.143422. 10.1016/j.scitotenv.2020.143422 33189377

[42] Li H, Dai S, Ouyang Z, Xie X, Guo H, Gu C et al. Multi-scale temporal variation of methane flux and its controls in a subtropical tidal salt marsh in eastern China. Biogeochemistry [Internet]. 2018;137(1–2):163–79. 10.1007/s10533-017-0413-y.

[43] Wei S, Han G, Chu X, Song W, He W, Xia J et al. Effect of tidal flooding on ecosystem CO2 and CH4 fluxes in a salt marsh in the Yellow River Delta. Estuar Coast Shelf Sci. 2020;232(May 2019).

[44] Rosentreter JA, Maher DT, Erler DV, Murray RH, Eyre BD (2018). Methane emissions partially offset blue carbon burial in mangroves. Sci Adv.

[45] Krithika K, Purvaja R, Ramesh R (2008). Fluxes of methane and nitrous oxide from an Indian mangrove. Curr Sci.

[46] Cameron C, Hutley LB, Friess DA, Brown B. High greenhouse gas emissions mitigation benefits from mangrove rehabilitation in Sulawesi, Indonesia. Ecosyst Serv [Internet]. 2019;40(November):101035. 10.1016/j.ecoser.2019.101035.

[47] Arifanti VB. Carbon Dynamics Associated with Land Cover Change in Tropical Mangrove Ecosystems of the Mahakam Delta, East Kalimantan, Indonesia. Oregon State University; 2017.

[48] Raich JW, Schlesinger WH (1992). The global carbon dioxide flux in soil respiration and its relationship to vegetation and climate. Tellus.

[49] Bouillon S, Connolly RM, Lee SY (2008). Organic matter exchange and cycling in mangrove ecosystems: recent insights from stable isotope studies. J Sea Res.

[50] Alongi DM. The energetics of Mangrove forests. Springer; 2009.

